# Reductions to main meal portion sizes reduce daily energy intake regardless of perceived normality of portion size: a 5 day cross-over laboratory experiment

**DOI:** 10.1186/s12966-020-0920-4

**Published:** 2020-02-12

**Authors:** Ashleigh Haynes, Charlotte A. Hardman, Jason C. G. Halford, Susan A. Jebb, Bethan R. Mead, Eric Robinson

**Affiliations:** 1 Centre for Behavioural Research in Cancer, Cancer Council Victoria, VIC 3004, Melbourne, Australia; 2 Department of Psychological Sciences, University of Liverpool, L69 7ZA, Liverpool, UK; 3 Nuffield Department of Primary Care Health Sciences, University of Oxford, Oxford, England

**Keywords:** Portion size, Obesity, Norms, Food environment, Food portion, Downsizing

## Abstract

**Background:**

Smaller portions may help to reduce energy intake. However, there may be a limit to the magnitude of the portion size reduction that can be made before consumers respond by increasing intake of other food immediately or at later meals. We tested the theoretical prediction that reductions to portion size would result in a significant reduction to daily energy intake when the resulting portion was visually perceived as ‘normal’ in size, but that a reduction resulting in a ‘smaller than normal’ portion size would cause immediate or later additional eating.

**Methods:**

Over three 5-day periods, daily energy intake was measured in a controlled laboratory study using a randomized crossover design (*N* = 30). The served portion size of the main meal component of lunch and dinner was manipulated in three conditions: ‘large-normal’ (747 kcal), ‘small-normal’ (543 kcal), and ‘smaller than normal’ (339 kcal). Perceived ‘normality’ of portion sizes was determined by two pilot studies. Ad libitum daily energy intake from all meals and snacks was measured.

**Results:**

Daily energy intake in the ‘large-normal’ condition was 2543 kcals. Daily energy intake was significantly lower in the ‘small-normal’ portion size condition (*mean* difference − 95 kcal/d, 95% CI [− 184, − 6], *p =* .04); and was also significantly lower in the ‘smaller than normal’ than the ‘small-normal’ condition (*mean* difference − 210 kcal/d, 95% CI [− 309, − 111], *p* < .001). Contrary to predictions, there was no evidence that the degree of additional food consumption observed was greater when portions were reduced past the point of appearing normal in size.

**Conclusions:**

Reductions to the portion size of main-meal foods resulted in significant decreases in daily energy intake. Additional food consumption did not offset this effect, even when portions were reduced to the point that they were no longer perceived as being normal in size.

**Trial registration:**

Prospectively registered protocol and analysis plan: https://osf.io/natws/; retrospectively registered: https://clinicaltrials.gov/ct2/show/NCT03811210.

## Introduction

Larger portions of food promote greater energy intake relative to smaller portions [1, 2]. A large number of studies suggest that intake is poorly adjusted following manipulation of external environmental factors, including portion size (see Levitsky [3] for a review). This has prompted calls to modify aspects of the food environment, including reducing the portion size of commercially available foods as part of policies to tackle obesity [4–7]. Previous research has demonstrated that reducing portion sizes decreases total meal intake in adults and children, but little research has examined decreases to daily energy intake [8–13]. In one controlled feeding study participants were provided with food to consume ad libitum over a 2-day period in standard or reduced portion sizes. Participants did not fully compensate for the smaller portion sizes by eating more food, leading to an overall reduction in daily energy intake in the ‘reduced’ relative to the ‘standard’ portion size condition [10]. However, not all evidence is entirely consistent with the view that consumers fail to adjust for reductions to food portion sizes. In a 6 month free-living study, participants were provided with boxed lunches in large, typical, or reduced portions [14]. While each reduction resulted in a decrease in lunch intake and the reduction from ‘large’ to ‘typical’ resulted in a significant reduction in self-reported total daily energy intake, there was no significant reduction in self-reported total daily energy intake in the ‘reduced’ relative to the ‘typical’ portion size condition. This suggests that participants may have responded to the reduced size lunch meal by eating more at other meals (but intake at other meals was not adjusted in response to the ‘larger’ portion size lunch). Therefore, whether or not consumers adjust their intake of other foods in response to portion size reductions may vary depending on the size of the reduction, and there is a need to understand how much portion sizes can be reduced by without causing consumers to engage in substantial additional eating of other food.

One potential determinant of the point at which reductions to portion size result in additional eating to compensate for the reduction is whether a reduced portion is visually perceived as being ‘normal’ in size. We recently proposed a ‘norm range model’ that posits that for most foods, there is a relatively wide range of portion sizes that are visually perceived by most people as being ‘normal’ and if portion size is reduced beyond this range additional eating may be likely to occur. We demonstrated that portions falling within that range are intended to be consumed in full without additional intake, but portions smaller than this result in intended additional food intake [15]. In two subsequent acute feeding studies, there was preliminary evidence that reducing the portion size of a main meal component to the point where it was perceived as ‘smaller than normal’ resulted in increased intake of other food during the meal (immediate additional intake) compared to the same sized reduction that resulted in the main meal component being perceived as ‘normal’ [9]. This additional intake did not completely offset the reduction in portion size: total energy intake from the meal was still significantly decreased regardless of whether the reduced portion size was perceived as ‘normal’ or not. However, it is possible that additional eating in response to reduced portion size in one meal may occur at subsequent eating occasions. Systematically examining whether consumers compensate for changes to portion size is vital for evaluating the overall effect of portion size reductions, and will be useful in informing effective portion size reduction strategies [16].

The present laboratory experiment is the first to investigate the effect of reducing the portion size of main meal components of lunch and evening meals (dinner) across three portion size conditions differing in perceived normality (perceived by an independent sample as ‘large-normal’, ‘small-normal’, ‘smaller than normal’) on daily energy intake over 5 days. In line with the proposed ‘norm range’ model, we predicted that *immediate* additional intake when the main meal was ‘small-normal’ would not be significantly different from when it was ‘large-normal’ in size, but there would be a significant increase in immediate additional intake when a ‘small-normal’ portion was reduced to ‘smaller than normal’. As the effect of reduced portion size on overall intake is only partially offset by additional eating of other foods [9], we expected a significant reduction in total energy intake at the portion size manipulated meal with each reduction to portion size. Critically, we predicted that there would be a significant reduction in total daily energy intake when portion sizes were reduced from ‘large normal’ to ‘small normal’, but not when reducing portion sizes from ‘small normal’ to ‘smaller than normal’, as over the course of a full day we reasoned that participants may be motivated to offset the reduced intake from portion sizes perceived as being ‘smaller than normal’ in size by eating other food.

## Methods

### Participants

**Fig. 1:**
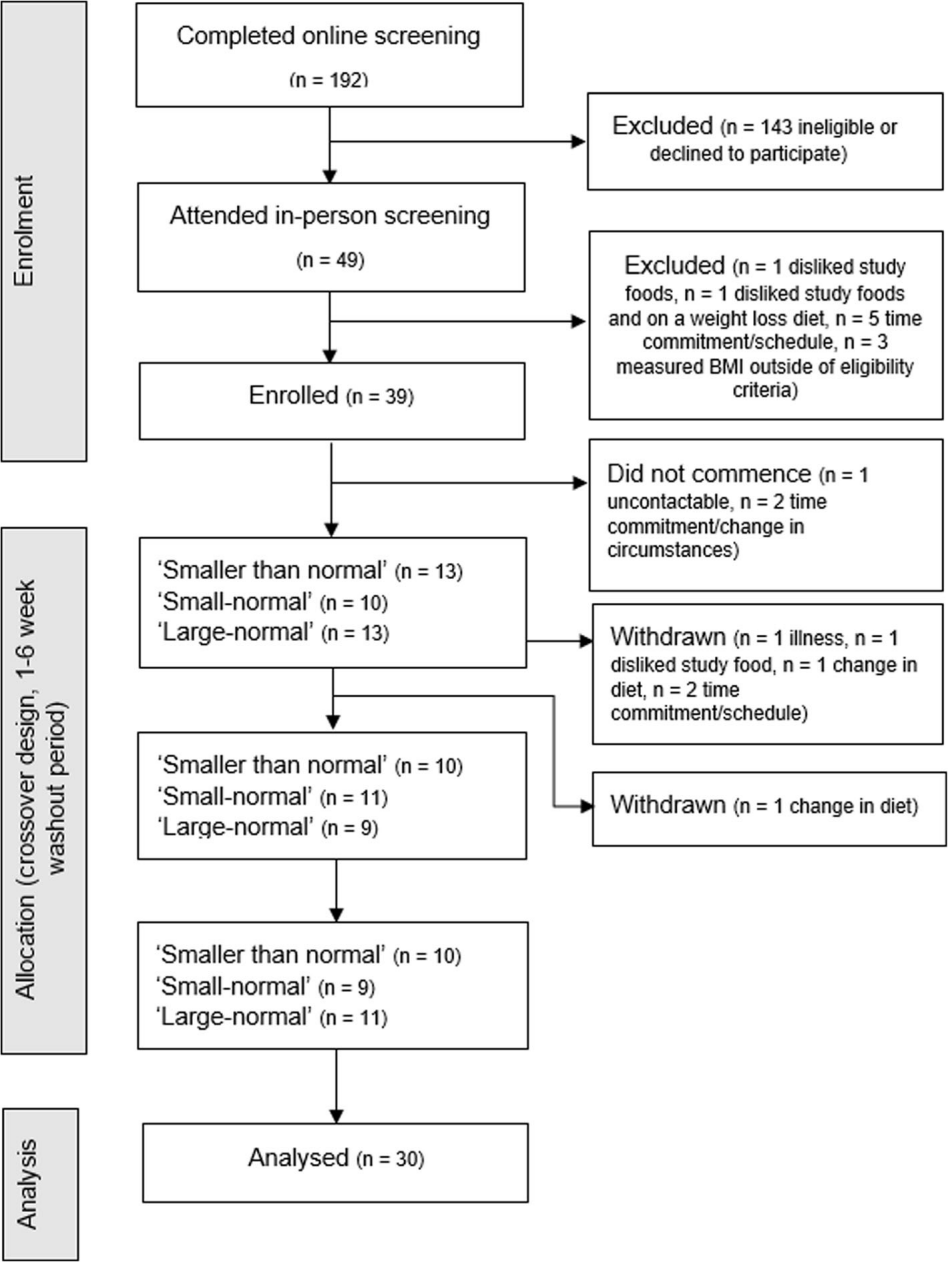
CONSORT Flow diagram depicting flow of participants through study recruitment, enrolment, completion, and analysis

Participants were recruited via social media, university announcements, flyers posted around campus and the local community, and direct emailing of research participation lists. Recruitment communication described the study as a ‘Daily mood and lifestyle study’. Individuals were eligible to participate if they were aged between 18 and 60 years; BMI 22.5 to 32.5 kg/m^2^; (as approximately 70% of adults in England have a BMI within this range; [17]); no food allergies, intolerances or specific dietary requirements (including being vegetarian or vegan); not currently dieting; no history of eating disorders; not taking medication which affected appetite; and willing to consume each of the test foods. Individuals who had participated in a portion size study in the past 12 months, or a weight loss trial in the past 4 weeks were ineligible to participate. These exclusion criteria were assessed by consulting a database of research participation and were confirmed verbally during the screening session, where participants were queried and asked to describe their previous research participation in a way that did not directly mention ‘portion size’. We aimed to recruit a sample with equal representation of participants in four categories stratified by gender and BMI category (lower BMI band: 22.5–27.5 kg/m^2^, higher BMI band: 27.5–32.5 kg/m^2^). Eligibility was assessed using an online questionnaire and confirmed during an in-person screening session (which included measurement of height and weight). See Fig. 1 for CONSORT flow diagram.

### Design

The portion size of the main components of lunch and dinner were manipulated in a randomized crossover design with three conditions (‘smaller than normal’, ‘small-normal’, and ‘large-normal’). Energy intake from all meals and snacks were measured in an eating behaviour laboratory over 3 (condition) × 5 day (Monday – Friday) testing periods separated by a washout period of 1–6 weeks. In each condition, participants were served three meals in the laboratory, were provided with a box of snacks to consume each day, and were requested not to consume any additional food apart from the items provided as part of the study. Participants were allocated to the sequence of conditions using a block-randomization pattern (see Additional file 1).

### Power calculation

In two previous studies examining the effect of portion size on energy intake [9], main effects of portion size on additional energy intake (after a portion-manipulated meal) were η_ρ_^2^ = 0.22, 0.36 (for the studies respectively), and on total energy intake (portion-manipulated meal plus additional intake) were η_ρ_^2^ = 0.32, 0.36. Assuming a minimum effect size of η_ρ_^2^ = .22, we determined that a sample of 20 was required for 80% power with an alpha level of .05 (G*Power 3.1) to detect a main effect of portion size on additional intake at lunch or dinner using 3 (portion size: ‘smaller than normal’, ‘small-normal’, ‘large-normal’) × 5 (test day: Monday – Friday) repeated measures ANOVAs. We aimed to recruit a minimum sample of 24 (to account for participant attrition and to allow full counterbalancing of condition sequence and equal representation of participants across gender and weight status groups) and a maximum sample of 30 completers if time permitted (to increase power to 95%).

### Study food

**Fig. 2:**
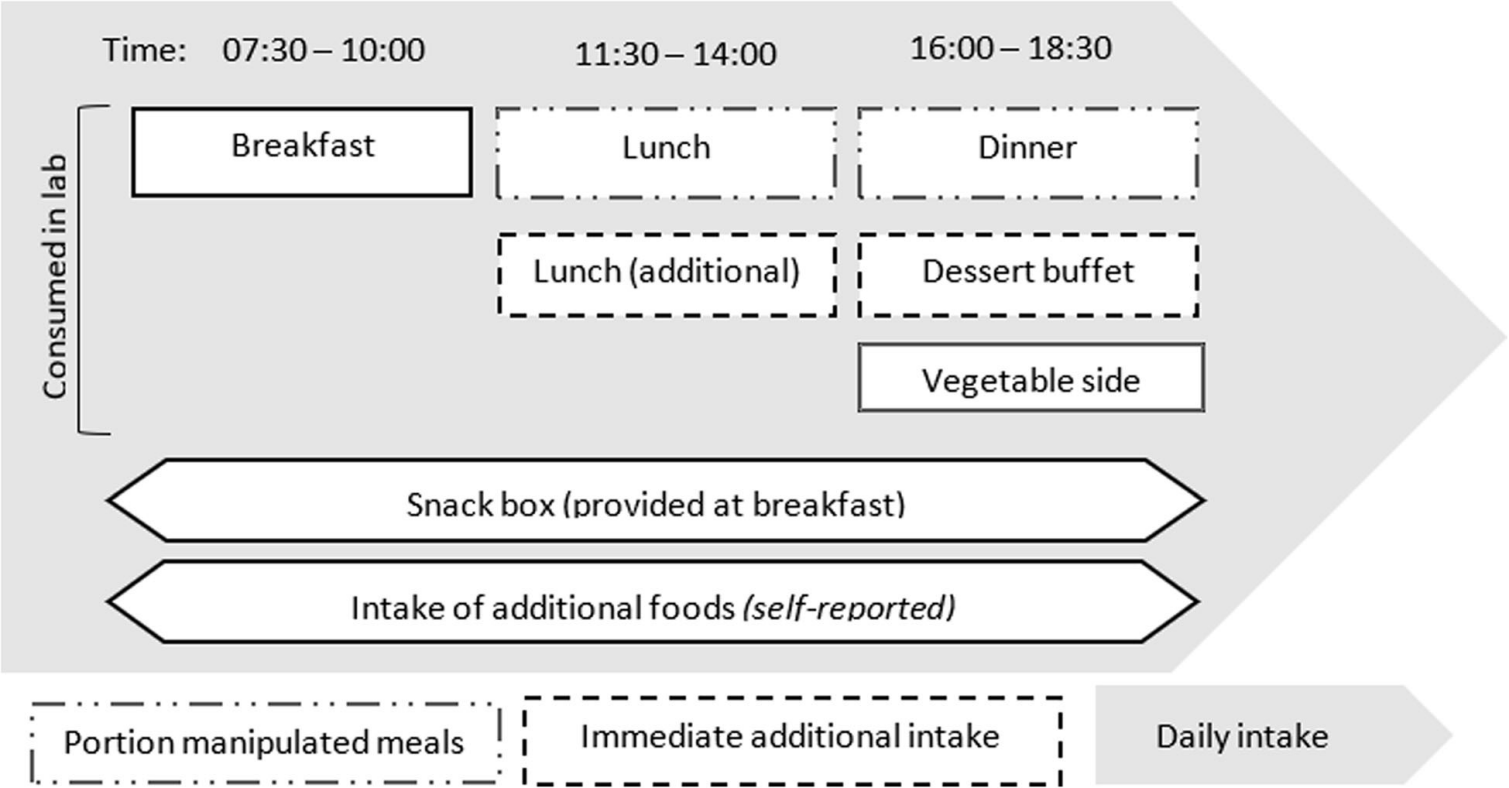
Overview of daily assessment of energy intake

See Fig. 2 for an overview of the assessment of energy intake. The size of the initial portion of the main component of lunch and dinner were manipulated; ‘large-normal’ *m* = 747 kcal, ‘small-normal’ *m* = 543 kcal, ‘smaller than normal’ *m* = 339 kcal. Selection of the portion sizes for lunch and dinner were informed by two pilot studies conducted on independent samples to identify (a) the range of portions of each meal perceived as ‘normal’ by the majority of a sample of adults viewing photographs in a computerised task, and then (b) to confirm the perception of portion sizes as ‘smaller than normal’, ‘small-normal’, and ‘large-normal’ when viewed in person, and to assess liking (Additional file 1). All other foods were provided in the same amount between conditions. Ad libitum breakfast, snacks, lunch (including second servings if required), and dinner with a vegetable side and dessert buffet were provided (see Additional file 1: Table S1 for full study menu, energy content and portion sizes). To be directly comparable with two recent studies examining immediate additional food intake in response to reductions to portion size [9], participants could serve themselves ad libitum additional helpings of the portion size manipulated food offered at lunch and ad libitum dessert following a fixed portion size manipulated meal at dinner. Each meal was served with 500 mL chilled water, and a choice of tea or coffee was offered at breakfast. Breakfast and snack box items were the same each day, and daily menus of a pasta dish (lunch), dish with rice accompaniment (dinner), and a dessert buffet (dinner) were presented on rotation throughout the testing period (in the same sequence for each participant and across testing periods). Each daily combination of lunch, dinner, and dessert buffet was matched as closely as possible on total energy content. Each food item provided to participants was prepared and served according to a standardised procedure. See Additional file 1 for further detailed information on meal components and selection of portion sizes.

### Procedure

To disguise the true purpose of the study [18], participants were told that the study aimed to investigate daily fluctuations in mood after accounting for lifestyle factors such as diet, activity, and sleep. Participants provided informed consent during an in-person screening session and attended the laboratory for breakfast, lunch, and dinner each day of the testing periods. Meal items that were designated as ‘immediate additional food intake’ (additional helpings of the portion size manipulated food consumed at lunch, dessert consumed at dinner) were placed on a serving tray behind participants’ seated position, and participants were instructed to serve themselves if they desired. All other meal components (breakfast, initial portion of main components of lunch and dinner) were served by placing a tray in front of participants while seated at a table, and all meal components (including additional intake items) were served simultaneously. A snack box for each day was provided to participants after they had consumed breakfast and was returned by participants at breakfast the following day to allow calculation of intake. Participants ate each laboratory meal alone in a quiet testing room without a time limit and were explicitly told that they were not required to finish any of the meal components.

Before and after each meal served in the laboratory, participants completed computerised mood, hunger and fullness ratings, and completed a computerised mood ‘filler’ task after lunch each day to bolster the cover story (see Additional file 1). Participants also completed a paper-pencil daily sleep questionnaire each morning to distract from the focus on food intake (e.g., “what time did you go to sleep last night?”, “what time did you wake up this morning?”). Data from mood and sleep measures were not analysed. Outside of the laboratory meals, participants were requested to drink tea, coffee, and soft drinks as they usually would during testing periods, not to drink in excess of 2 alcoholic drinks per day, and not to consume any additional food apart from the items provided as part of the study. Participants were requested not to consume anything apart from water after midnight on the Sunday preceding each testing period which began on Monday. Participants’ weight was measured by a researcher after breakfast on Monday, and after dinner on Friday of each testing period (which were separated by a washout period of 1–6 weeks).

Participants attended a final session on the Monday or Tuesday following completion of the third testing period to complete a computerized portion size normality rating task, report what they though the aims of the study were, and to complete additional questionnaires on demographic and eating-related variables not related to the pre-registered hypotheses of this study (see Additional file 1). Finally, participants were debriefed and provided with financial compensation. The study was conducted between February and December 2018 and was conducted in line with the Helsinki Declaration and institutional ethical approval (IPHS 2688). The study protocol and analysis plan were pre-registered on the Open Science Framework (https://osf.io/natws/), and is registered on clinicaltrials.gov (https://clinicaltrials.gov/ct2/show/NCT03811210).

### Measures

#### Study-provided energy intake

Energy intake from food consumed in the laboratory and from the snack box was assessed by multiplying consumption weights (measured to the nearest 0.01 g using a digital scale [Sartorius]) by manufacturer-provided energy density values for each meal component.

#### Out of study energy intake

Participants were requested not to eat any food apart from the meals and snacks provided by the research team during each testing period, but to record the amount and type of any extra-study food they did consume in a paper-pencil diary. Participants were requested to complete an entry in the diary as soon as possible after having consumed the extra food, and the diary was returned each morning. A daily total of out of study energy intake was calculated from participant diaries using myfood24 [19, 20].

#### Moderate-vigorous physical activity (MVPA)

Participants wore an activity monitor (Fitbit Zip™) continuously during each testing period (except while swimming and bathing) to assess MVPA (operationalised as minutes of activity with a metabolic equivalent [MET] of ≥3). Fitbit device estimates of MVPA have been validated against gold standard research-grade physical activity monitoring devices, and data from the devices have good reliability [21–23].

#### Discretionary leisure-time physical activity (LTPA)

Participants completed part 4 of the International Physical Activity Questionnaire (IPAQ, wording modified to refer to a 5 day testing period) on the Monday following each testing period. An estimate of discretionary LTPA was calculated (MET-minutes = physical activity in minutes * MET level [walking = 3.3, moderate intensity = 4.0, vigorous intensity = 8.0] * 5 days). Although the validity of the IPAQ against objective measures of physical activity is limited, the measure has acceptable reliability making it suitable for assessing within-person changes in activity in crossover designs [24, 25].

#### Hunger and fullness

Hunger and fullness was assessed using computerised visual analog scales ranging from 0 (‘not at all’) to 100 (‘extremely’), which were embedded in a series of mood ratings to bolster the cover story and detract from a focus on eating (e.g., “how angry do you currently feel?”). Daily hunger and fullness ratings across time were summarised by calculating the area under the curve using the trapezoid function [26].

#### Height and weight

Height was measured during the screening session using a stadiometer (Seca) to the nearest 0.5 cm and weight was measured during the screening session, after breakfast on Monday and after dinner on Friday of each testing period, using a digital scale (Salter) to the nearest 0.1 kg. Measurements were taken without shoes and heavy outer clothing.

#### Portion size normality (manipulation check)

Perceived normality of each portion size was assessed at the end of the study in a computerised task programmed in Psychopy [27]. Participants viewed a picture of each portion size of the portion-manipulated lunch and dinner dishes and asked: “In your opinion, how normal is this portion? By ‘normal’ we mean whether the portion contains a normal amount of food to eat for a single meal.” Responses were indicated on 7-point Likert scales ranging from 1 (*not normal, it is far too small*), to 7 (*not normal, it is far too big*), with a midpoint of 4 (*normal).*

#### Awareness of study aims

At the end of the study participants were asked to report what they thought were the aims of the study. See Additional file 1 for full details.

### Analysis plan

#### Primary analyses

All statistical analyses were conducted in IBM SPSS 24.0. The primary dependent measures were (a) daily energy intake (sum of energy intake from all laboratory meals, snack box, and self-reported additional energy intake) (b) immediate additional intake (energy intake from self-served additional helpings of lunch, self-served dessert at dinner), (c) total main meal intake (sum of energy intake from main component and additional intake at lunch, dinner). All primary dependent measures were compared between portion size conditions in separate repeated-measures ANOVAs with a 3 (portion size condition) × 5 (day: Monday to Friday) design. Analyses of measures (b) and (c) included an additional 2-level ‘meal’ factor (lunch, dinner). We hypothesised that the reduction from ‘large-normal’ to ‘small-normal’ would result in a significant reduction to overall daily intake (a) but the reduction from ‘small normal’ to ‘smaller than normal’ would not. In line with previous findings [9], we predicted that immediate additional intake (b) would not significantly differ between the ‘small-normal’ and ‘large-normal’ conditions, but would be significantly greater in the ‘smaller than normal’ condition than the ‘small-normal’ condition. Also in line with previous findings, we predicted that total main meal intake (c) would be significantly reduced in the ‘small-normal’ relative to the ‘large-normal’ portion size condition, and in the ‘smaller than normal’ relative to the ‘small-normal’ portion size condition. In our primary pre-registered analyses alpha was set at 0.05.

Sensitivity analyses were conducted to examine whether the pattern of results from the primary analyses (a) to (c) differed after firstly, excluding participants who were aware of the true aims of the study (to examine the effect of portion size on primary or secondary outcomes [intake, physical activity, appetite] or on primary outcomes only [intake]), and secondly, excluding outliers on main outcome variables (identified as those with a value >3SD from condition mean) and influential cases (identified as those with a Cook’s distance of > 1, indicating a multivariate outlier) [28]. As decided a priori*,* data from these participants are included in the reported analyses, and the significance of primary results did not vary depending on their inclusion unless otherwise stated. We also examined whether the pattern of results was dependent on the order in which portion size conditions were presented (see Additional file 1).

#### Portion size normality

To test whether the reduced main meal portion sizes were perceived as ‘smaller than normal’, and ‘normal’ as intended, we conducted a series of one-sample *t*-tests on normality ratings for each of the foods, with a test value of 4 (equal to perceived ‘normal’ in response to “how normal do you think this portion size is”). We predicted that the perceived normality rating for the ‘smaller than normal’ portion size would be significantly lower than 4, while the normality ratings for the ‘small-normal’ portions would not significantly differ from 4 at a Bonferroni-corrected alpha of 0.004.^1^ As planned a priori, we present mean normality ratings for the ‘large-normal’ portions but only made predictions regarding the perceived normality of the two reduced portions, as they constitute the manipulation of interest (“reduction of portion size to ‘normal’ or to ‘smaller than normal’”).

#### Bodyweight stability

To analyse stability of weight between each testing period and throughout the study, participant bodyweight was compared between portion size conditions using a 3 (condition) × 2 (time: start, end of week) repeated-measures ANOVA.

#### Secondary analyses

We conducted a series of analyses to assess the impact of portion size manipulation on secondary outcome measures (hunger and fullness, objective MVPA, self-reported discretionary LTPA). Exploratory analyses examined the effect of portion size condition on breakfast, snack box, and out of study energy intake. Main effects across secondary and exploratory analyses were evaluated against an alpha of *p* < 0.0167 to correct for multiple analyses.

## Results

### Sample characteristics

Thirty-nine participants were enrolled in the study after completing online and in-person eligibility screening. Nine enrolled participants either did not commence the study or were withdrawn, leaving a final sample of *N* = 30. See Table 1 for sample characteristics and Fig. 1 for CONSORT participant flow diagram.

**Table 1:** Sample characteristics (*N* = 30)

	*N* (%) / *M* (*SD*), range
Gender	15 (50% female)
Age	31.6 (10.3), 18–56
Education	
High school	3 (10%)
Some university	2 (7%)
Bachelor’s degree	11 (37%)
Master’s degree	10 (33%)
Doctoral or professional degree	4 (13%)
BMI	26.0 (2.3), 22.5–29.8
Weight status	
Normal weight	11 (37%)
Overweight	19 (63%)
Restrained eating score	2.61 (0.56), 1.40–3.50^a^

^a^ Scale bounds: 1–5

### Effect of portion size on daily energy intake

**Fig. 3:**
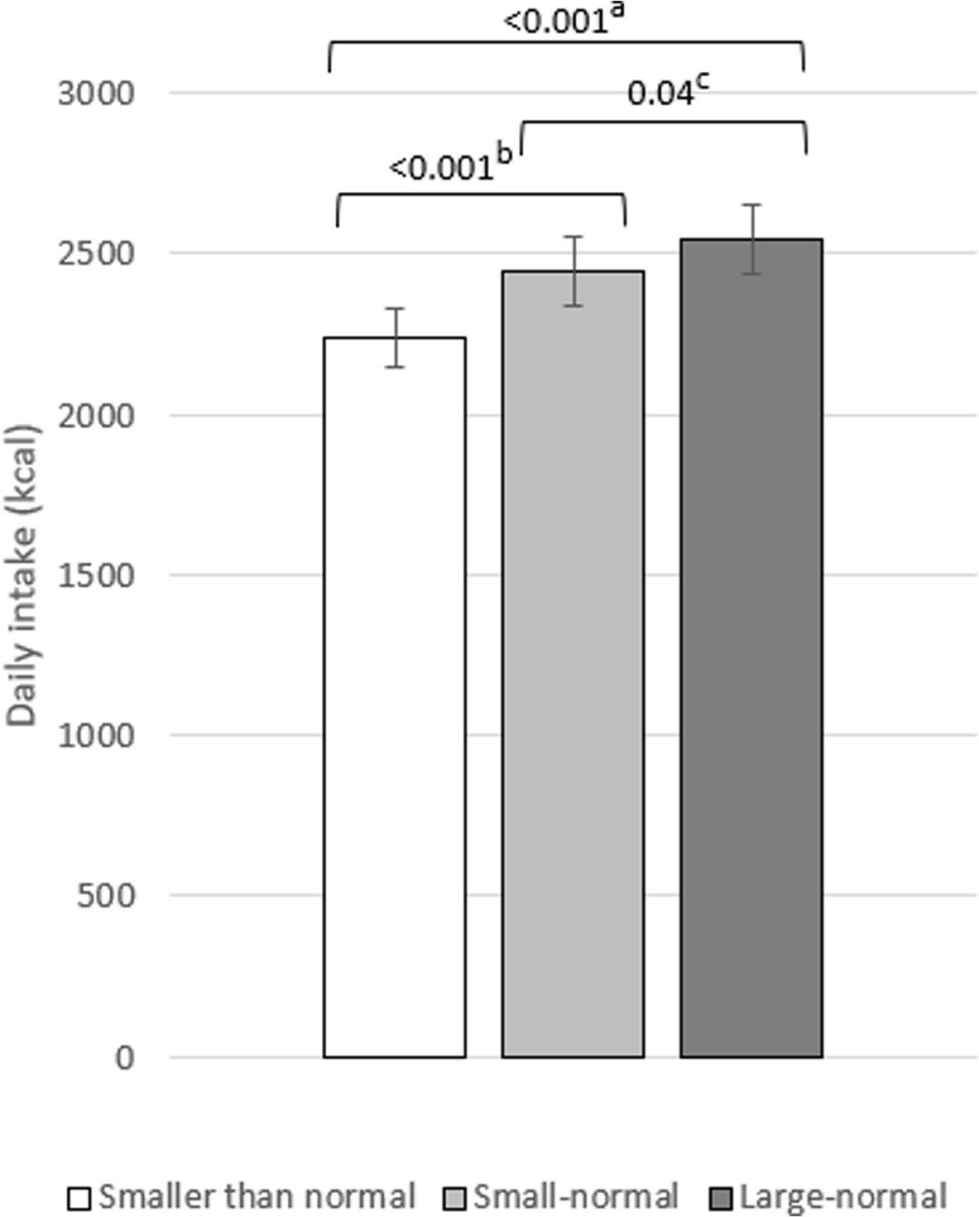
Effect of portion size on daily energy intake. ^a^ 95% CI [− 418, − 192], *d =* 1.01. ^b^ 95% CI [− 309, − 111], *d =* 0.79. ^c^ 95% CI [− 184, − 6], *d =* 0.40. Error bars represent standard errors and values on comparison bars = *p* for pairwise comparisons

Portion size condition had a significant effect on daily energy intake and there was no significant interaction between portion size condition and day (see Table 2 for means, Table 3 for ANOVA results, and Additional file 1 for energy intake plotted by day). Mean daily energy intake was highest in the ‘large-normal’ condition (2543 kcals, *sd* = 592) and contrary to predictions, each reduction to portion size was associated with a significant reduction to daily energy intake^2^ (Fig. 3).

**Table 2:** Mean energy intake, bodyweight, physical activity, and hunger and fullness ratings by portion size condition (*SD*)

	‘Smaller than normal’	‘Small-normal’	‘Large-normal’
*Total energy provided (kcal/d)*	*5074*	*5485*	*5897*
Daily energy intake (kcal/d)	2238 (490)	2448 (584)	2543 (592)
Breakfast	441 (154)	441 (175)	429 (174)
Lunch (total)	653 (203)	695 (221)	768 (210)
Portion	313 (13)	501 (56)	658 (97)
Immediate additional intake	339 (196)	194 (190)	110 (140)
Dinner (total)	613 (162)	752 (181)	851 (214)
Portion	338 (22)	486 (79)	628 (119)
Immediate additional intake	275 (154)	266 (147)	223 (149)
Snack box	454 (229)	452 (239)	428 (218)
Out of study intake (self-reported)	78 (91)	108 (126)	68 (80)
Body weight (kg, Monday)	77.0 (10.8)	76.6 (10.9)	76.8 (10.9)
Body weight (Friday)	76.9 (11.0)	76.69 (10.6)	77.1 (11.0)
MVPA (mins/day)^a^	70.2 (44.0)	75.8 (42.0)	74.9 (51.6)
MVPA (mins/day) ^b^	72.2 (8.0)	71.3 (3.8)	70.0 (3.9)
Discretionary LTPA (MET mins/week)	1024.5 (907.7)	1003.0 (985.1)	1207.5 (1046.8)
Hunger ^c^	310.3 (104.8)	288.9 (104.0)	284.5 (112.8)
Fullness ^c^	432.8 (84.8)	424.0 (79.7)	432.8 (97.2)

All *n* = 30 except ^a^ complete cases, *n* = 20. ^b^ Estimated marginal means and *SE* from multiply-imputed datasets. ^c^ Area under curve of meal ratings across the day. MVPA = FitBit measured moderate to vigorous physical activity with MET ≥3. Discretionary LTPA = leisure time physical activity from self-report. Immediate additional intake refers to additional helpings of the portion size manipulated food consumed at lunch and dessert consumed at dinner

**Table 3:** ANOVA results: portion size effect on primary intake variables

Dependent variable	Main effect portion size	Interaction ^a^
Daily energy intake	*F*(2, 58) = 20.09, *p* < 0.001, η_ρ_^2^ = 0.41	*F*(5.50, 159.60) = 0.70, *p* = 0.64, η_ρ_^2^ = 0.02
Immediate additional intake	*F*(1.68, 48.60) = 52.72, *p* < 0.001, η_ρ_^2^ = 0.65	*F*(2, 58) = 30.16, *p* < 0.001, η_ρ_^2^ = 0.51 ^b^
Lunch	*F*(1.57, 45.47) = 65.29, *p* < 0.001, η_ρ_^2^ = 0.69	*F*(5.22, 151.28) = 1.84, *p* = 0.11, η_ρ_^2^ = 0.06
Dinner	*F*(2, 58) = 6.09, *p* = 0.004, η_ρ_^2^ = 0.17	*F*(4.66, 135.07) = 0.61, *p* = 0.68, η_ρ_^2^ = 0.02
Total meal intake	*F*(1.54, 44.64) = 50.89, *p* < 0.001, η_ρ_^2^ = 0.64	*F*(1.56, 45.30) = 13.12, *p* < 0.001, η_ρ_^2^ = 0.31 ^b^
Lunch	*F*(2, 58) = 17.83, *p* < 0.001, η_ρ_^2^ = 0.38	*F*(5.48, 159.04) = 0.88, *p* = 0.50, η_ρ_^2^ = 0.03
Dinner	*F*(1.52, 44.13) = 51.96, *p* < 0.001, η_ρ_^2^ = 0.64	*F*(4.89, 141.80) = 0.56, *p* = 0.73, η_ρ_^2^ = 0.02

^a^ All interactions portion x day, except ^b^ interaction portion x meal (lunch, dinner). Immediate additional intake refers to additional helpings of the portion size manipulated food consumed at lunch and dessert consumed at dinner

### Effect of portion size on immediate additional intake

**Fig. 4:**
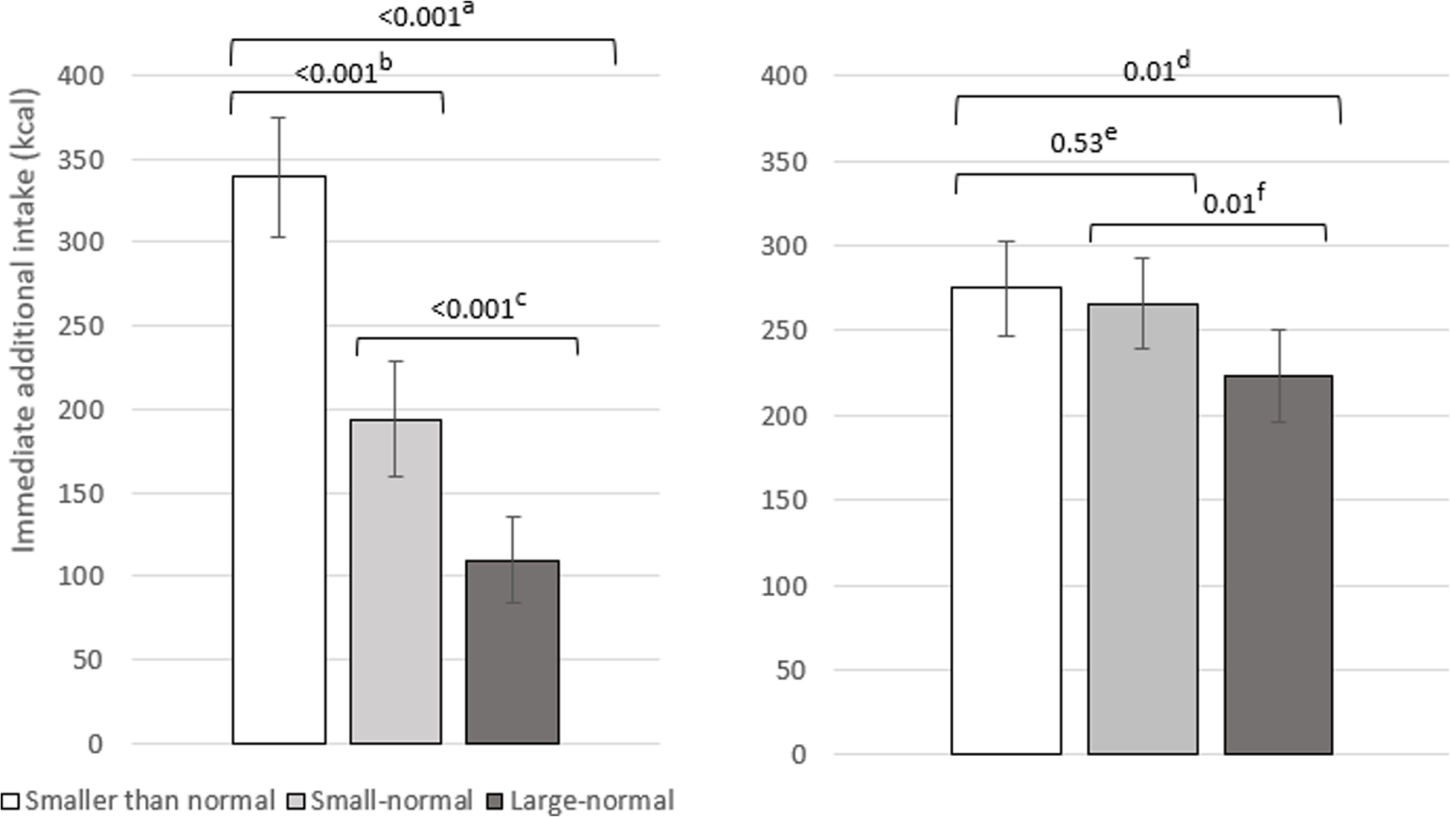
Effect of portion size on immediate additional intake of other meal food at lunch (left) and dessert food at dinner (right). ^a^ 95% CI [179, 281], *d =* 1.69. ^b^ 95% CI [106, 184], *d =* 1.40. ^c^ 95% CI [51, 117], *d =* 0.95. ^d^ 95% CI [15, 88], *d =* 0.53. ^e^ 95% CI [− 20, 38], *d* = 0.12. ^f^ 95% CI [12, 73], *d =* 0.53. Error bars represent standard errors and values on comparison bars = *p* for pairwise comparisons

There was a significant effect of portion size condition on immediate additional intake at lunch and dinner (combined), and a significant interaction between condition and meal (see Table 3 for ANOVA results). In separate portion size condition x day repeated-measures ANOVAs for lunch and dinner, portion size condition significantly affected immediate additional intake, with no significant interaction between portion size and day. Immediate additional intake after lunch was smallest in the ‘large-normal’ condition (*mean* = 110 kcal, *sd* = 140) and contrary to predictions, each subsequent reduction to portion size was associated with a significant and similar increase in immediate additional intake at lunch (Fig. 4). Immediate additional intake after dinner was smallest in the ‘large-normal’ condition (*mean* = 223 kcal, *sd* = 149) and contrary to predictions, was significantly larger in the ‘small-normal’ condition than the ‘large-normal’ condition, but did not significantly differ between the ‘smaller than normal’ and ‘small-normal’ conditions. Thus, there was no evidence that additional energy intake was lower when reduced portions appeared ‘normal’ than when they appeared ‘smaller than normal’.

### Effect of portion size on total main meal intake

**Fig. 5:**
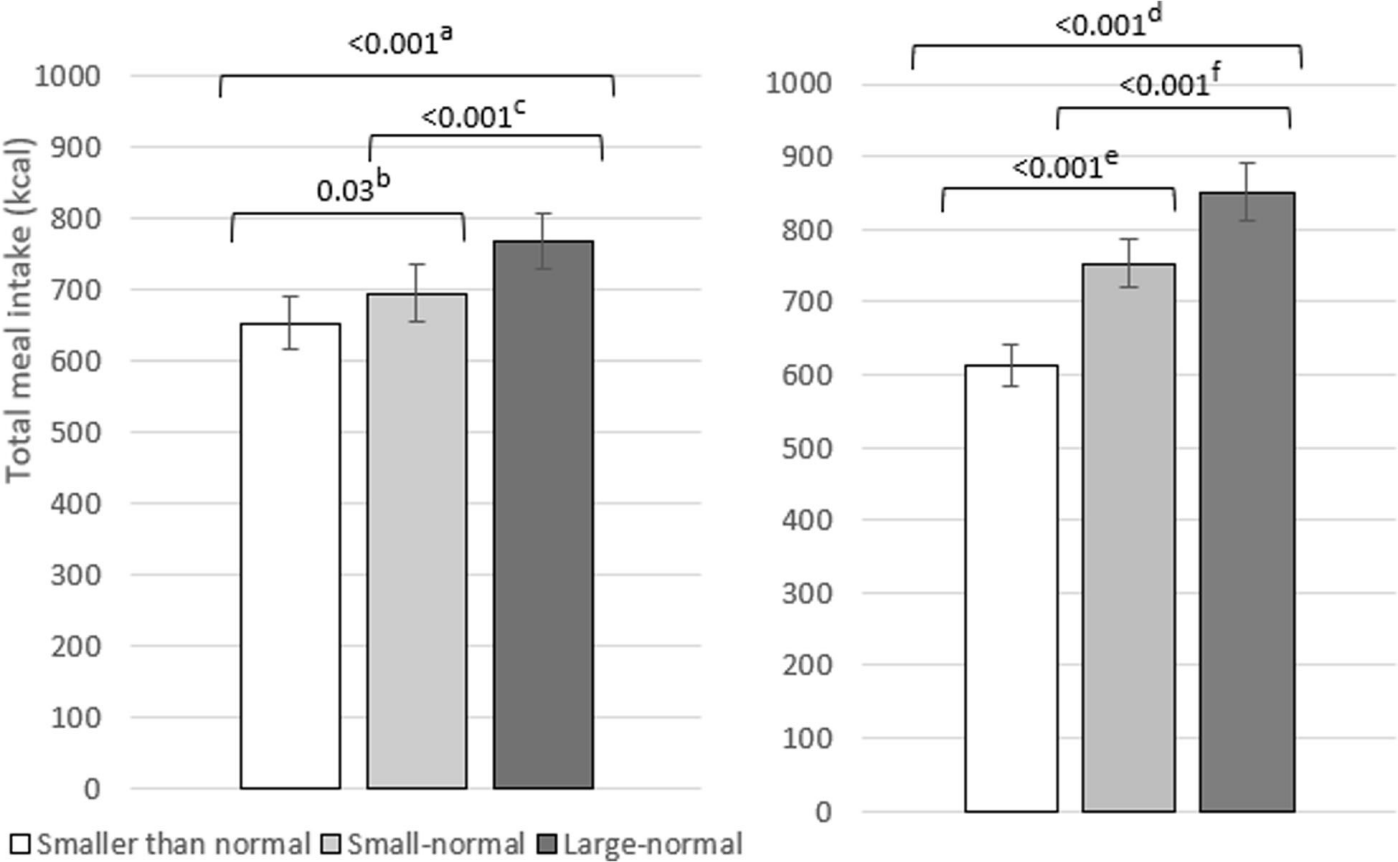
Effect of portion size on total meal intake (sum of intake from initial portion and additional intake of other meal food) at lunch (left), and (sum of intake from initial portion and additional intake of dessert food) at dinner (right). ^a^ 95% CI [− 163, − 68], *d =* 0.92. ^b^ 95% CI [− 82, − 4], *d =* 0.41. ^c^ 95% CI [− 106, − 40], *d =* 0.84. ^d^ 95% CI [− 299, − 179], *d =* 1.49. ^e^ 95% CI [− 178, − 100], *d* = 1.32. ^f^ 95% CI [− 141, − 57], *d =* 0.88. Error bars represent standard errors and values on comparison bars = *p* for pairwise comparisons

There was a significant effect of portion size on total main meal intake (lunch and dinner combined), and a significant interaction between portion size condition and meal (see Table 3 for ANOVA results). In separate portion size condition x day repeated-measures ANOVAs for lunch and dinner, portion size condition significantly affected total meal intake, with no significant interaction between portion size condition and day. Mean total lunch intake (768 kcals, *sd* = 210) and mean total dinner intake (851 kcals, *sd* = 214) was highest in the ‘large-normal’ condition and each reduction to portion size was associated with a significant and similar sized reduction to total lunch intake and total dinner intake (Fig. 5).

### Analysis of order effects

We examined the pattern of results across groups according to the sequence in which participants received the portion size conditions and found little evidence that condition sequence affected the results of the main analyses. There was a significant interaction between condition sequence and portion size condition for total lunch meal energy intake. However, controlling for condition sequence did not alter the significance of pairwise comparisons between portion size conditions and the pattern of results was largely consistent across condition sequences (see Additional file 1).

### Perceived normality of portion sizes

As predicted, ‘smaller than normal’ portions of each dish were rated as significantly smaller than the midpoint of the scale (all *p* < 0.001, indicating a significant deviation from a rating of perceived ‘normal’). Also as predicted, ratings of the ‘small normal’ portion sizes of most dishes did not significantly differ from the midpoint of the perceived normality scale (indicating that these portions were perceived as being relatively ‘normal’ as intended), with the exception of beef curry and chili con carne, where normality ratings of the ‘small normal’ portion sizes were slightly higher than the scale midpoint. In an additional analysis suggested by an anonymous reviewer, we found that normality ratings of the ‘large-normal’ portions were significantly larger than the scale midpoint (Table S4, Additional file 1).

### Additional analyses

There were no significant effects of portion size condition on hunger or fullness, daily moderate to vigorous physical activity, discretionary leisure-time physical activity, or body weight (see Table 2 for descriptive statistics, and Additional file 1: Table S2 for full ANOVA results). In exploratory analyses, neither breakfast, snack box, nor out of study (self-reported) energy intake significantly varied between portion size conditions (Table 2; Additional file 1: Table S2). The study was not designed or powered to detect moderation by individual differences, but the pattern of daily energy intake across conditions was consistent across gender and BMI groups (see Additional file 1: Figure S4).

## Discussion

Reducing the portion size of lunch and dinner meals resulted in a significant reduction to daily energy intake across 5 days. Based on a ‘norm range’ model of portion size, we predicted that a reduction that resulted in portions appearing ‘smaller than normal’ in size would invite substantial additional energy intake and this would result in no overall decrease to daily energy intake, but there was no evidence of this. Rather, the results of the present study suggest that reductions to the portion size of main-meal foods result in significant decreases in daily energy intake regardless of the perceived normality of portion size. Even reductions to portion size that are noticeable and result in portions that appear small lowered daily energy intake.

The results of the present study are not fully consistent with some of the results from two acute single meal studies [9]. In these previous studies, a reduction that resulted in a portion size being perceived as ‘smaller than normal’ resulted in an increase in immediate additional energy intake, while a reduction that resulted in a portion that was still perceived as ‘normal’ in size, did not. However, in the previous studies [9], each reduction to the portion size of the main meal component resulted in a significant reduction in total meal intake at both lunch and dinner. Additional eating only partially made up for the difference in intake from the initial portion. This was also observed in the present study. Therefore, unlike findings from virtual and short-term food intake studies [9, 15], results of the present study are not consistent with a norm range model of portion size, as reducing portions past the point of perceived normality did not significantly alter (via additional eating) the influence portion size had on daily energy intake. It may be the case that when food intake is examined over longer periods of time, cognitive appraisals like perceived normality of portion size may have a smaller influence on additional eating behaviour than in the short-term [9]. An alternative explanation for our findings and the influence of portion size is that any portion size served acts as a form of normative anchor [29] that then biases consumers’ decision about how much to eat, which would explain why in the present study even portions that were reduced so much that they appeared ‘smaller than normal’ reduced daily energy intake and why even increases from large to very large portion sizes can drive energy intake upwards [2].

In the only other controlled laboratory-based feeding study of reducing portion sizes we are aware of, portion sizes of all foods provided were reduced by 25% (or by 821–1076 kcal) [10]. Here we manipulated only the main meal component at lunch and dinner while all other foods were provided in sufficient quantities allowing additional eating to make up for the smaller portion sizes, resulting in a 412 kcal/d decrease in food served with each portion size reduction. A sizable proportion of the reduction to portion size at main meals was transferred to overall energy intake. For example, the reduction from the ‘small-normal’ to the ‘smaller than normal’ portion (412 kcal/day reduction in food provided across two meals) resulted in a reduction to intake from those meals of 327 kcal/day, and an overall reduction in total daily energy intake of 210 kcals. These findings are consistent with the results of a systematic review which demonstrated that energy deficits imposed by experimental manipulation are poorly compensated for [30]. A potential argument against reducing portions of commercially available food products is that consumers may compensate through additional eating for the reduced portions which may result in no overall benefit to total energy intake [31]. However the findings of the present study suggest that reductions to the portion sizes of commercially available foods may effectively reduce energy intake, regardless of whether reduced portion sizes are perceived as ‘normal’ or not [31].

A strength of the present research is that energy intake from all food and drink provided was objectively measured over a 5 day period. This represents the longest controlled laboratory-based study on the effect of *reducing* portion size (relative to standard portion sizes) of which we are aware [10], although a previous study examined the effect that *very large* portions have on energy intake in the laboratory over 11 days [31]. The present research also presents the first examination of whether energy intake over 5 days in response to reduced portions differs depending on whether a reduced portion is perceived as ‘normal’ in size or not. The selection of ‘smaller than normal’, ‘small normal’, and ‘large normal’ portion sizes for each dish was informed by the results of a pilot study in an independent sample of participants. This approach was adopted to minimise hypothesis awareness among the study participants that would likely have been compromised by completing pre-study ratings of portion size normality. While the post-study manipulation check results confirmed that all ‘smaller than normal’ portions were perceived as such by participants and the majority of the ‘small-normal’ portions were rated as normal, two of six were statistically significantly larger than ‘normal’, although ratings were still close to ‘normal’ on the scale. These slight differences may be attributable to the manipulation check methodology. Manipulation check data may have been contaminated by repeated exposure to and consumption of the dishes during the study [32, 33]. The manipulation checks for each portion size were also administered consecutively and this may have artificially produced larger differences between the portion size conditions.

Although we requested that participants not consume food outside of the study, we accounted for any energy intake consumed outside of the laboratory using self-reported food diaries. This self-reported intake may be subject to some underreporting, but we presume this would be similar across conditions. A limitation is that most participants in the present study had at least some university education. A different pattern of results may have been observed with a more representative sample, as evidence from an online study suggests that portion size influences intended food consumption to a greater extent among individuals with lower education [34]. Energy intake was examined in response to three manipulated portion sizes. Examination of energy intake from a wider range of portion sizes will be useful to identify the point at which reduced portions trigger significant compensatory behaviour and to enable testing of other potential mechanisms that could determine this point. We examined energy intake over five days as this is feasible in a laboratory setting. Despite decreasing overall energy intake across the 5 days, the portion size reductions did not produce an increase in appetite or a decrease in physical activity. Given that we found no evidence of adaptions to reduced portion size by the end of 5 days in our study and the effect that *larger* portions have on daily energy intake has been observed up to 11 days [35], in line with Rogers and Brunstrom [36] we presume that longer-term compensation would only be expected in response to weight loss and not merely in response to a lower energy balance [37, 38]. All study foods were standardised across testing periods and were neutral to moderately liked by participants, meaning that additional intake is unlikely to have been unduly affected by dislike of study foods. However, as is the case with any laboratory-based experiment, responses to manipulations of portion size may differ in free-living settings. The influence of portion size has also been demonstrated outside of the confines of the laboratory [11, 14, 39–42], however in one free living study a reduction from a ‘standard’ to a ‘reduced’ portion size lunch was not associated with a significant reduction in overall daily energy intake [14]. The artificial environment imposed in controlled laboratory-based experiments (including but not limited to the provision of a limited number of free foods) may impact on energy intake and the extent to which compensation for reduced portion sizes occurs. Replication in real-world settings would now be informative.

## Conclusions

Reductions to the portion size of main-meal foods resulted in significant decreases to daily energy intake, even when portions were reduced to the point that they were no longer perceived as being normal in size. Even relatively large reductions to portion size that are noticeable and result in portions that appear small are still likely to lower total energy intake.

## Supplementary information

**Additional file 1.Supplementary Information:** Additional methodological information, full study menu, additional analyses and results.

## Data Availability

The datasets supporting the conclusions of this article are available in the Open Science Framework repository (https://osf.io/natws/).

## Abbreviations

BMI: Body mass index
IPAQ: International physical activity questionnaire
kcal: Kilocalories
LTPA: Leisure time physical activity
MET: Metabolic equivalent of task
MVPA: Moderate to vigorous physical activity

## Footnotes

1 Our pre-registered analysis strategy was to evaluate one-sample *t-*tests against an alpha of *p <* 0.0028. Here we used an adjusted alpha of *p <* 0.004 instead, as we evaluated only 12 pairwise comparisons as planned (*p =* 0*.*05 / 12).

2 When participants who were aware of the primary (the effect of portion size on intake) or secondary aims of the study (the effect of portion size on physical activity or appetite) (*n* = 4), or when participants who were aware of the primary aims (*n* = 3) were excluded, the pattern of results was consistent with the main analysis. Each reduction to portion size resulted in a reduction to energy intake, except that the ‘large-normal’ to ‘small-normal’ reduction was not statistically significant (*p* = .08, 95% CI [− 187, 12], *d =* 0.28; *p* = .05, 95% CI [− 191, 2], *d* = 0.39, respectively).

## References

[1] Hollands GJ, Shemilt I, Marteau TM, Jebb SA, Lewis HB, Wei Y, Higgins, JPT, Ogilvie D. Portion, package or tableware size for changing selection and consumption of food, alcohol and tobacco. Cochrane Database Syst Rev. 2015;(9):CD011045. 10.1002/14651858.CD011045.pub2. 10.1002/14651858.CD011045.pub2 PMC4579823 26368271

[2] Zlatevska N, Dubelaar C, Holden SS (2014). Sizing up the effect of portion size on consumption: a meta-analytic review. J Mark.

[3] Levitsky DA (2005). The non-regulation of food intake in humans: Hope for reversing the epidemic of obesity. Physiol Behav.

[4] Livingstone MBE, Pourshahidi LK (2014). Portion size and obesity. Adv Nutr: Int Rev J.

[5] Marteau TM, Hollands GJ, Shemilt I, Jebb SA. Downsizing: policy options to reduce portion sizes to help tackle obesity. Br Med J. 2015;351:h5863. 10.1136/bmj.h5863 26630968

[6] Nielsen SJ, Popkin BM (2003). Patterns and trends in food portion sizes, 1977-1998. J Am Med Assoc.

[7] Steenhuis I, Poelman M (2017). Portion size: latest developments and interventions. Curr Obes Rep.

[8] Lewis HB, Ahern AL, Solis-Trapala I, Walker CG, Reimann F, Gribble FM (2015). Effect of reducing portion size at a compulsory meal on later energy intake, gut hormones, and appetite in overweight adults. Obesity..

[9] Haynes A, Hardman CA, Halford JCG, Jebb SA, Robinson E. Portion size normality and additional within-meal food intake: two crossover laboratory experiments. Br J Nutr. 2019. 10.1017/S0007114519002307 31488225

[10] Rolls BJ, Roe LS, Meengs JS (2006). Reductions in portion size and energy density of foods are additive and lead to sustained decreases in energy intake. Am J Clin Nutr.

[11] Vermote M, Versele V, Stok M, Mullie P, D'Hondt E, Deforche B (2018). The effect of a portion size intervention on French fries consumption, plate waste, satiety and compensatory caloric intake: an on-campus restaurant experiment. Nutr J.

[12] Reale S, Kearney C, Hetherington M, Croden F, Cecil J, Carstairs S (2018). The feasibility and acceptability of two methods of snack portion control in United Kingdom (UK) preschool children: reduction and replacement. Nutr.

[13] Carstairs S, Caton S, Blundell-Birtill P, Rolls B, Hetherington M, Cecil J (2018). Can reduced intake associated with downsizing a high energy dense meal item be offset by increased vegetable variety in 3–5-year-old children?. Nutr.

[14] French SA, Mitchell NR, Wolfson J, Harnack LJ, Jeffery RW, Gerlach AF (2014). Portion size effects on weight gain in a free living setting. Obes.

[15] Haynes A, Hardman CA, Makin ADJ, Halford JCG, Jebb SA, Robinson E (2019). Visual perceptions of portion size normality and intended food consumption: a norm range model. Food Qual Prefer.

[16] Halford J. C. G., Masic U., Marsaux C. F. M., Jones A. J., Lluch A., Marciani L., Mars M., Vinoy S., Westerterp-Plantenga M., Mela D. J. (2018). Systematic review of the evidence for sustained efficacy of dietary interventions for reducing appetite or energy intake. Obesity Reviews.

[17] NatCen Social Research (2016). Health Survey for England, 2014. Department of Epidemiology and Public Health University College London.

[18] Robinson Eric, Bevelander Kirsten E., Field Matt, Jones Andrew (2018). Methodological and reporting quality in laboratory studies of human eating behavior. Appetite.

[19] Albar SA, Alwan NA, Evans CEL, Greenwood DC, Cade JE (2016). Agreement between an online dietary assessment tool (myfood24) and an interviewer-administered 24-h dietary recall in British adolescents aged 11–18 years. Br J Nutr.

[20] Carter MC, Albar SA, Morris MA, Mulla UZ, Hancock N, Evans CE (2015). Development of a UK online 24-h dietary assessment tool: myfood24. Nutr.

[21] Evenson KR, Goto MM, Furberg RD (2015). Systematic review of the validity and reliability of consumer-wearable activity trackers. Int J Behav Nutr Phys Act.

[22] Imboden MT, Nelson MB, Kaminsky LA, Montoye AH. Comparison of four Fitbit and Jawbone activity monitors with a research-grade ActiGraph accelerometer for estimating physical activity and energy expenditure. Br J Sports Med. 2017; Epub 2017/05/10. 10.1136/bjsports-2016-096990 28483930

[23] Kooiman TJM, Dontje ML, Sprenger SR, Krijnen WP, van der Schans CP, de Groot M. Reliability and validity of ten consumer activity trackers. BMC Sports Sci, Med Rehabil. 2015;7(1):24. 10.1186/s13102-015-0018-5 PMC4603296 26464801

[24] Craig CL, Marshall AL, Sjostrom M, Bauman AE, Booth ML, Ainsworth BE (2003). International physical activity questionnaire: 12-country reliability and validity. Med Sci Sports Exerc.

[25] Lee PH, Macfarlane DJ, Lam T, Stewart SM (2011). Validity of the international physical activity questionnaire short form (IPAQ-SF): a systematic review. Int J Behav Nutr Phys Act.

[26] Pruessner JC, Kirschbaum C, Meinlschmid G, Hellhammer DH (2003). Two formulas for computation of the area under the curve represent measures of total hormone concentration versus time-dependent change. Psychoneuroendocrinology.

[27] Peirce JW. Psychophysics software in Python. J Neurosci Methods. 2007;162(1–2):8–13. 10.1016/j.jneumeth.2006.11.017 PMC2018741 17254636

[28] Stevens JP (1984). Outliers and influential data points in regression analysis. Psychol Bull.

[29] Marchiori D, Papies EK, Klein O (2014). The portion size effect on food intake. An anchoring and adjustment process?. Appetite.

[30] Levitsky David A., Sewall Anna, Zhong Yingyi, Barre Laura, Shoen Stefan, Agaronnik Nicole, LeClair Jean-Luc, Zhuo Wendy, Pacanowski Carly (2019). Quantifying the imprecision of energy intake of humans to compensate for imposed energetic errors: A challenge to the physiological control of human food intake. Appetite.

[31] Public Health England. Calorie reduction: the scope and ambition for action. 2018; Available from: https://www.gov.uk/government/publications/calorie-reduction-the-scope-and-ambition-for-action. Accessed 28 Jan 2020.

[32] Robinson Eric, Kersbergen Inge (2018). Portion size and later food intake: evidence on the “normalizing” effect of reducing food portion sizes. The American Journal of Clinical Nutrition.

[33] Robinson Eric, Henderson Jodie, Keenan Gregory S., Kersbergen Inge (2019). When a portion becomes a norm: Exposure to a smaller vs. larger portion of food affects later food intake. Food Quality and Preference.

[34] Best M, Papies EK (2019). Lower socioeconomic status is associated with higher intended consumption from oversized portions of unhealthy food. Appetite.

[35] Rolls BJ, Roe LS, Meengs JS (2007). The effect of large portion sizes on energy intake is sustained for 11 days. Obesity (Silver Spring).

[36] Rogers Peter J., Brunstrom Jeffrey M. (2016). Appetite and energy balancing. Physiology & Behavior.

[37] Hall KD, Heymsfield SB, Kemnitz JW, Klein S, Schoeller DA, Speakman JR (2012). Energy balance and its components: implications for body weight regulation. Am J Clin Nutr.

[38] Hall KD (2010). Predicting metabolic adaptation, body weight change, and energy intake in humans. Am J Physiol-Endocrinol Metab.

[39] Best Maisy, Barsalou Lawrence W., Papies Esther K. (2018). Studying human eating behaviour in the laboratory: Theoretical considerations and practical suggestions. Appetite.

[40] Hollands GJ, Cartwright E, Pilling M, Pechey R, Vasiljevic M, Jebb SA, Marteau TM. Impact of reducing portion sizes in worksite cafeterias: a stepped wedge randomised controlled pilot trial. Int J Behav Nutr Phys Act. 2018;15(78). 10.1186/s12966-018-0705-1 PMC6097301 30115084

[41] Jeffery Robert W, Rydell Sarah, Dunn Caroline L, Harnack Lisa J, Levine Allen S, Pentel Paul R, Baxter Judith E, Walsh Ericka M (2007). Effects of portion size on chronic energy intake. International Journal of Behavioral Nutrition and Physical Activity.

[42] Diliberti N, Bordi PL, Conklin MT, Roe LS, Rolls BJ (2004). Increased portion size leads to increased energy intake in a restaurant meal. Obes Res.

