# Supplementary material for: Reductions to main meal portion sizes reduce daily energy intake regardless of perceived normality of portion size: a 5 day cross-over laboratory experiment
Source: Int J Behav Nutr Phys Act. 2020 Feb 12;17:21. doi: 10.1186/s12966-020-0920-4 (PMC7017560; doi:10.1186/s12966-020-0920-4)
Supplement: Supplementary file 1 — Additional file 1.Supplementary Information: Additional methodological information, full study menu, additional analyses and results. [file 12966_2020_920_MOESM1_ESM.pdf]

Reductions to main meal portion sizes reduce daily energy intake regardless of perceived normality of portion size: a 5 day cross-over laboratory experiment

Supplementary Information

Table of contents

Additional methodological information……………………………………………………...page 3

Full study menu ……………………………………………………………………….....page 10

Additional analyses and results………...…………………………………………………...page 13

Additional methodological information

Counterbalancing

Participants were pseudo-randomly assigned to receive the portion size conditions in one of six possible sequences. Randomisation was achieved using 'RANDBETWEEN' (Microsoft Excel) to generate a random number between 1 and 6, with a block-randomisation structure such that in each block of six participants, one participant was assigned to each of the sequences resulting in an equal number of participants assigned to each sequence. Participants were also randomised to one of the 120 possible sequences of the 5 versions of the computerised mood filler measure across the 5 test days, using the same approach. Participants received the same sequence of computerised mood tasks across the three testing periods (conditions), such that the only difference between testing periods was the portion size of lunch and dinner dishes.

Selection of portion sizes

The portion-manipulated test meals consist of a pasta-based dish for lunch (penne with pesto, spaghetti Bolognese, spaghetti carbonara), and a dish served with rice for dinner (beef curry, chicken korma curry, chilli con carne). To select the dishes and portion sizes for these meals, we conducted two pilot studies. For the first pilot study, a selection of 7 candidate main meal dishes (3 pasta lunchtime dishes: penne with pesto, spaghetti carbonara, spaghetti Bolognese; 4 dinner dishes: beef curry, chicken korma curry, chilli con carne, sweet and sour chicken [all served with rice]) were photographed on standard-sized (28.5cm diameter) dinner plates at portion sizes ranging from 40 to 300% of a reference portion (equal to the manufacturer's recommended serving for commercially available foods, or equivalent to similar commercially available foods for recipes prepared from ingredients). Participants (N = 30, 50%

female) completed a computer-based rating task in which they viewed each portion size in a randomised order and indicated whether they perceived that portion as a ‘normal’ or ‘not normal’ amount to eat. We calculated the proportion of the sample that perceived each portion size as ‘normal’ in size to determine the ‘norm range’ for each dish (i.e., the range of portions perceived as ‘normal’ by at least 60% of the sample). The ‘normal’ portion size for each food was selected from the lower end of the norm range, the ‘large normal’ portion size from the mid-high end of the norm range, and the ‘smaller than normal’ portion size from below the norm range. Each portion size reduction (from ‘large-normal’ to ‘small-normal’, and from ‘small-normal’ to ‘smaller than normal’) for each food represented a reduction of 178-223 kcal.

In a second pilot study, 10 participants (50% female) viewed each main meal dish in the three portion sizes in person. Participants rated the perceived normality of each portion for each dish on a Likert scale ranging from 1 (“not normal – too small”) to 7 (“not normal – too large”), with a midpoint of 4 (“normal”). Participants subsequently tasted each dish and rated how much they liked the taste on a 7-point Likert scale ranging from 1 (“dislike very much”) to 7 (“like very much”). All dishes were at least moderately liked by all participants, such that only a small number of participants rated their liking of the meals below the midpoint of the scale (maximum $n = 2$ for any one dish), and the mean liking rating was above the midpoint of the scale for all dishes (m liking for each rice dish significantly > 4, m liking of each pasta dish > 4.5 but did not significantly differ from 4). The mean normality ratings of all ‘smaller than normal’ portion sizes were significantly below the midpoint of the scale (rating of 4, corresponding to perceived ‘normal’), and the maximum normality rating across all dishes was 3, suggesting that the ‘smaller than normal’ portions were reliably perceived as such. For most dishes, the mean ratings of the ‘small-normal’ portion sizes did not significantly differ from the midpoint of the scale, and the

majority (at least 60%) of participants provided a normality rating of '4' for most dishes indicating that participants perceived these portions as 'normal'. There were two exceptions: the mean normality rating for the 'small-normal' portion of pasta with pesto was significantly higher than 4 (indicating perceived 'larger than normal'), and the mean normality rating for the 'small-normal' portion of chicken with sweet and sour sauce was significantly lower than 4 (indicating perceived 'smaller than normal'). To ensure that all 'small-normal' portions were indeed perceived as at least normal in size, we decided to omit sweet and sour chicken with rice from the menu. This resulted in a final menu of 3 lunchtime pasta dishes and 3 rice-based dinner dishes for rotation throughout each testing week (Table S1). The least-liked pasta dish (spaghetti Bolognese), and least liked rice dish (beef curry with rice) were chosen to be served only once in each weekly menu, with the other two pasta and rice dishes being served twice per week for all participants.

Selection of non-manipulated portion sizes

Quantities of additional served foods for *ad libitum* consumption (additional self-served lunch meal, dessert after dinner) were selected based on the quantities provided in previous laboratory-based studies, in which we observed that participants did not tend to finish the additional food (1). Servings for breakfast and snacks were determined by multiplying manufacturer-recommended portion sizes by 2-4 times. No participants requested extra food in addition to what was provided, suggesting that the amount of food provided was sufficient.

Computerised mood measure

As a filler task to bolster the cover story, participants completed a 1-minute lexical decision task after lunch each day. The task was described to participants as "a new way to assess

mood by measuring speed of responding to positive versus neutral words”. Five positive words, 5 neutral words, and 5 ‘non-words’ were presented on the computer screen one by one in a random order. Participants were asked to respond to each word/non-word by pressing the left or right key marked on the keyboard to indicate whether the task displays a word or non-word (according to the [counterbalanced] key assignment specified in the task instructions). There were 5 different versions of the task, each consisting of different word sets. Participants were randomised to a 5-day sequence of task versions, which was repeated across the three testing periods.

Awareness of study aims

Participants reported what they thought were the aims of the study in an open-ended response format as part of an online questionnaire programmed using Qualtrics. Responses that referred to the effect of portion size on intake, physical activity, or appetite, were coded as ‘aware of aims’. We also coded participants as ‘aware of primary aims’ if they referred to the effect of portion size on intake. Coding was completed independently by two researchers and there were no discrepancies.

Additional final session questionnaires

The following measures were administered in the final testing session to provide data for future research questions and exploratory analyses. We did not plan *a priori* to analyse the data from these measures in relation to our hypotheses about portion size in this study.

Manipulation awareness

To assess awareness of the difference in portion sizes between the testing periods, participants were asked a series of questions with a yes/no response format about their experience of the study. The questions consisted of several filler questions to distract from the focus on

portion size (e.g., “I noticed a difference in the mood measures I completed between the three testing periods”, “I noticed a difference in the taste of the foods between the three testing periods”), and a single item to assess awareness of the portion size manipulation (“I noticed a difference in the size of the lunch and dinner portions between the three testing periods”). If participants indicated being aware of the difference in portion sizes between testing periods, on the following page of the online questionnaire they were asked to order the portion sizes (smallest, medium, largest lunch and dinner portions) according to the testing period (1, 2, 3) in which they were served.

Awareness of the influence of portion size on intake

Participants were asked to indicate on a 7-point Likert scale ranging from 1 (*strongly disagree*) to 7 (*strongly agree*) the extent to which they agree that the portion size served for lunch and dinner on each day influenced how much they ate.

Usual portion size task

Participants completed a computerised task programmed in Psychopy in which they indicated the portion size of each of the portion-manipulated lunch and dinner foods that was closest to what they would usually serve themselves. The task began with the presentation of an image displaying a portion size equal to 40% of the reference portion. Participants adjusted the size of the displayed portion using the ‘up’ and ‘down’ arrow keys until it appeared equivalent to the amount that they would usually serve themselves, when they pressed ‘enter’ to select the portion size. Each arrow key press increased or decreased the portion by an increment of 10% of the reference portion, and the portion sizes presented range from 40 to 300% of the reference portion. This measure was repeated for each portion-manipulated lunch and dinner dish, with each dish presented in a random order.

Liking

Participants were asked to report how much they liked (from 1 [*not at all*], to 7 [*like it a lot*]), each of the specific foods presented in the study.

Restrained, external, and emotional eating.

Participants completed the Dutch Eating Behaviour Questionnaire (DEBQ; 2), which consists of 33 items in three subscales measuring restrained, external, and emotional eating tendencies. The scales have been demonstrated to have good internal reliability (2). Responses to the ten 5-point Likert restrained eating items were averaged to produce a restrained eating score ranging from 1-5.

Plate clearing tendency

A 5-item scale was used to assess participants' tendencies to clear their plate when eating (e.g., " I always clear my plate when eating"; 3). The scale has good internal reliability (3).

Satiety responsiveness

Participants completed the 4-item satiety responsiveness subscale of the Adult Eating Behaviour Questionnaire (e.g., "I often get full before my meal is finished") (4), in which they indicated their agreement with each statement on a 5-point Likert response scale ranging from 1 (strongly disagree) to 5 (strongly agree). The scale has been validated as a measure of appetitive traits in adults and has good internal consistency (4).

Self-control

The brief self-control scale was used to assess trait self-control (5). Participants responded to 13-items on 5-point Likert scales ranging from 1 (*strongly disagree*), to 5 (*strongly agree*) (e.g., "I am good at resisting temptation"). The scale has good internal reliability (5).

Dieting status

A single item was administered to assess whether participants are currently dieting (yes/no).

Attitudes toward food waste

Participants completed a 5-item self-report scale to assess attitudes toward food waste (e.g., “it is fine for food to go to waste sometimes”, 7-point Likert response: 1 [*strongly disagree*], to 7 [*strongly agree]*).

Table S1.
Full Study menu: portions provided, mean liking ratings

Food item	Portion (g)	Energy (kcal)	Mean liking (SD)
Breakfast [a]		1364	
White toast (4 slices)	161	392	5.8 (0.9)
Cornflakes	120	455	3.4 (1.7)
Margarine	40	169	
Strawberry jam	60	148	
Semi-skimmed milk	500	200	
Lunch			
Smaller than normal		*1413*	
Small-normal		*1630*	
Large-normal		*1847*	
Penne pasta pesto [b]			5.2 (1.7)
Smaller than normal	*188*	*335*	
Small-normal	*313*	*558*	
Large-normal	*438*	*781*	
Extra available	626	1115	
Spaghetti carbonara [b]			4.2 (2.1)
Smaller than normal	*224*	*318*	
Small-normal	*373*	*531*	
Large-normal	*522*	*743*	
Extra available	746	1061	
Spaghetti bolognese [c]			5.3 (1.3)
Smaller than normal	*228*	*324*	
Small-normal	*380*	*540*	
Large-normal	*532*	*755*	
Extra available	760	1079	
Dinner			
Smaller than normal		1498	
Small-normal		1692	

Large-normal		1887	
Chicken korma curry with rice [b]			6.0 (1.2)
Smaller than normal	*228*	*356*	
Small-normal	*358*	*560*	
Large-normal	*488*	*764*	
Beef curry with rice [c]			5.5 (1.1)
Smaller than normal	*228*	*312*	
Small-normal	*358*	*490*	
Large-normal	*488*	*668*	
Chilli con carne with rice [b]			5.2 (1.7)
Smaller than normal	*277*	*386*	
Small-normal	*415*	*580*	
Large-normal	*554*	*773*	
Mixed steamed vegetables [a]	80	50	
Flapjack bites (8) [b]	144	577	4.3 (1.7)
Chocolate cake bites (8) [b]	101	502	4.8 (1.3)
Caramel shortcake bites (8) [b]	118	595	5.0 (1.6)
Raspberry and vanilla cake rolls (12) [b]	105	499	5.2 (1.7)
Choc chip cake bites (10) [c]	104	545	4.8 (1.5)
Chocolate brownie bites (10) [c]	131	555	5.1 (1.4)
Snack box [a]		800	
Apples (2)	266	142	5.0 (1.8)
Chocolate fruit and nut mix	30	140	5.7 (1.4)
Crisps (2 packs)	36	198	
Cereal bars (2)	46	162	4.6 (2.0)
Biscuits (2)	24	116	5.1 (1.6)
Carrot sticks	100	42	4.2 (2.2)
Total			
Smaller than normal		5074	
Small-normal		5485	

Large-normal	5897

Note. 500mL water provided with each meal, and choice of tea or coffee and sugar with breakfast. For unit-based foods, number of pieces provided in parentheses (portion values are approximated for *n* pieces). Total values averaged across meal types for lunch and dinner. [a] identical each day, [b] served twice per week, [c] Served once per week. Commercial details for foods available at https://osf.io/natws/

Additional analyses and results

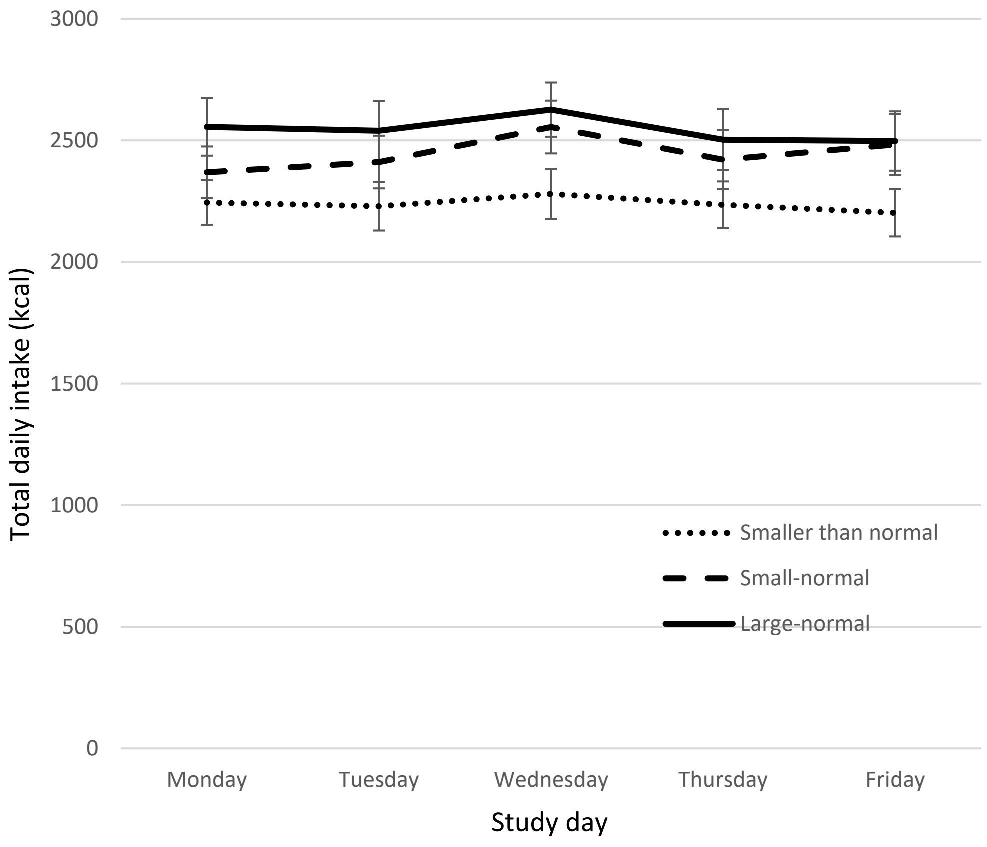

Figure S1 Energy intake (kcal) across conditions by day. No significant interaction portion size x day, or main effect of day. Error bars represent standard errors.

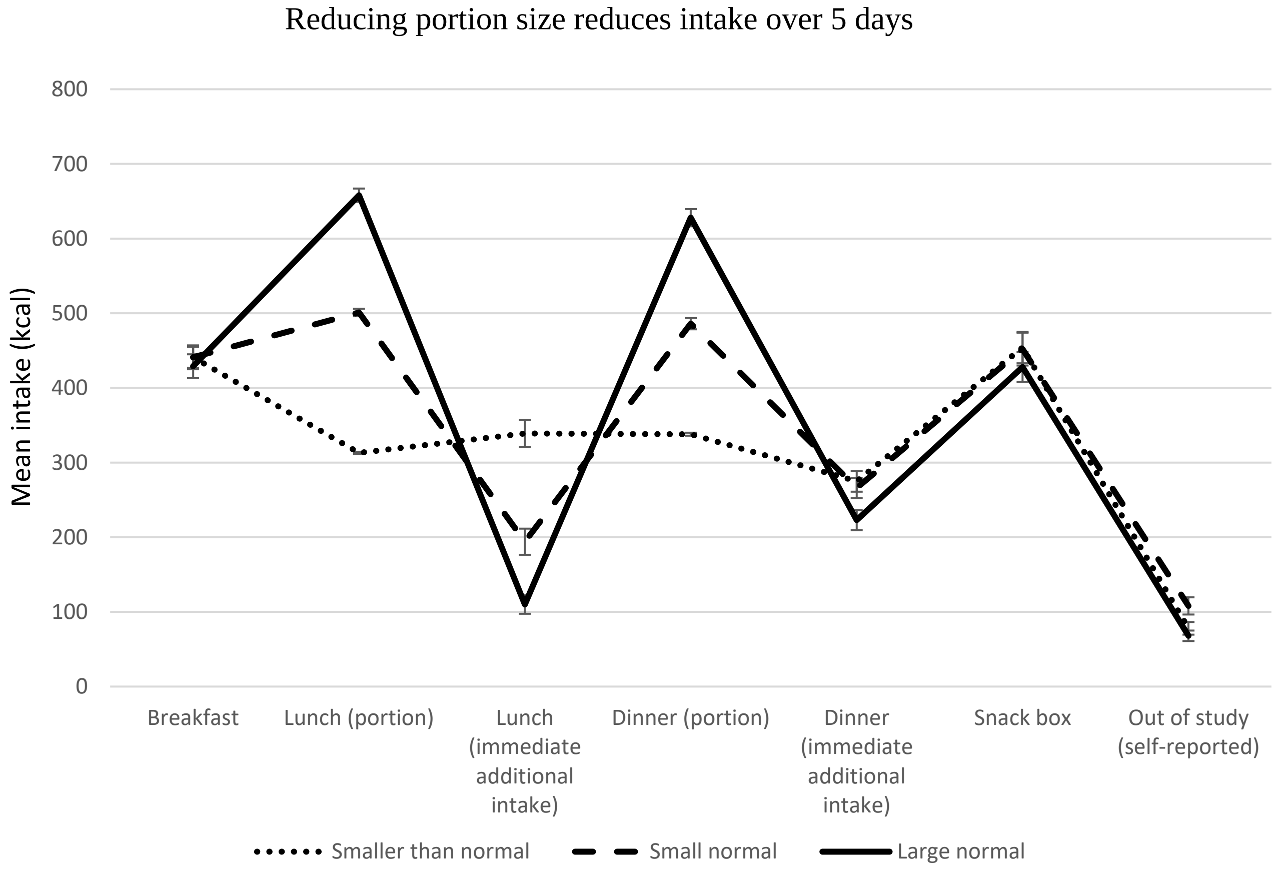

Figure S2 Energy intake (kcal) across conditions by menu component. Values are means (across days), error bars represent standard errors.

Table S2.

Full ANOVA results: portion size effect on secondary intake variables, body weight, physical activity, hunger and fullness.

Dependent variable	Main effect portion size	Interaction [a]
Breakfast intake	$F(2, 58) = 0.47, p = 0.63, \eta_\rho^2 = 0.02$	$F(8, 232) = 1.05, p = 0.40, \eta_\rho^2 = 0.04$
Snack box intake	$F(2, 58) = 0.96, p = 0.39, \eta_\rho^2 = 0.03$	$F(5.89, 170.91) = 1.10, p = 0.37, \eta_\rho^2 = 0.04$
Out of study intake	$F(2, 58) = 3.76, p = 0.03, \eta_\rho^2 = 0.12$	$F(3.88, 112.54) = 1.07, p = 0.37, \eta_\rho^2 = 0.04$
Body weight	$F(2, 58) = 2.20, p = 0.12, \eta_\rho^2 = 0.07$	$F(2, 58) = 0.97, p = 0.39, \eta_\rho^2 = 0.03$ [b]
MVPA (complete cases) [d]	$F(2, 38) = 0.49, p = 0.62, \eta_\rho^2 = 0.03$	$F(4.49, 85.21) = 0.85, p = 0.51, \eta_\rho^2 = 0.04$
Discretionary LTPA	$F(2, 28) = 0.37, p = 0.69, \eta_\rho^2 = 0.03$	*n/a*
Hunger and fullness	$F(2, 58) = 2.59, p = 0.08, \eta_\rho^2 = 0.08$	$F(2, 58) = 1.97, p = 0.15, \eta_\rho^2 = 0.06$ [c]

Note. All *p* values assessed against an adjusted alpha level of <.0167 for multiple comparisons (no main or interaction effects were statistically significant). [a] All interaction portion x day, except [b] Interaction portion x time (Monday, Friday) and [c] Interaction portion x rating (hunger, fullness). [d] *n* = 10 missing data due to loss of or failure to wear device; effect of portion size in linear mixed models analysis of multiply imputed data in SPSS with 5 imputed datasets, $F(2, 275.55) = 0.15$, $p = 0.86$.

Order effects

The primary analyses were also repeated with an additional 6-level between subjects factor (portion size sequence) to examine whether the pattern of results were dependent on the order in which portion size conditions were presented. As this analysis was only adequately powered to detect a statistically large effect of portion size sequence, we also plotted the primary analysis results by sequence group for visual inspection (Figures S2a to S2e). There was no significant interaction between portion size condition and the sequence in which conditions were completed in mixed ANOVA for any of the primary dependent variables, except for total meal intake for lunch (Table S2). In analyses controlling for portion size sequence, none of the pairwise comparisons differed from the results of primary analyses, suggesting that the results were not largely dependent on the sequence in which the portion size conditions were assigned.

Table S3.

Mixed ANOVA results: interaction portion size condition x portion sequence

Dependent variable	
Immediate additional intake (lunch)	$F = 1.61$, $\eta_\rho^2 = 0.25$
Immediate additional intake (dinner)	$F = 0.84$, $\eta_\rho^2 = 0.15$
Total meal intake (lunch)	$F = 2.11$, $\eta_\rho^2 = 0.31$*
Total meal intake (dinner)	$F = 0.90$, $\eta_\rho^2 = 0.16$
Daily intake	$F = 1.01$, $\eta_\rho^2 = 0.17$

*$p = 0.04$. All other $p > 0.13$.

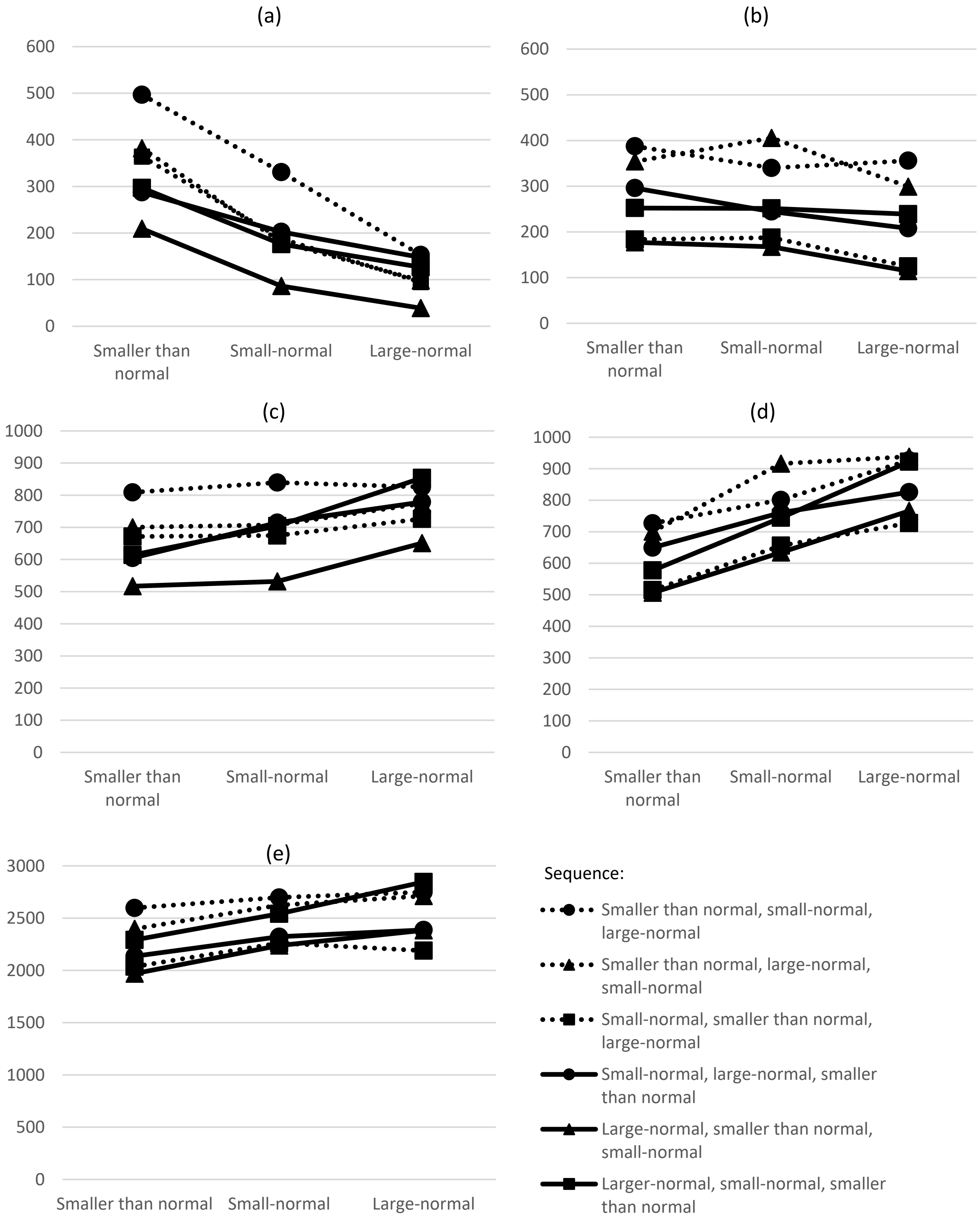

Figure S3. Energy intake (kcal) across conditions by sequence: (a) immediate additional intake lunch, (b) immediate additional intake dinner, (c) total intake lunch, (d) total intake dinner, (e) daily intake.

Effect of portion size by gender and BMI category

This study is underpowered to detect significant interactions between condition and either gender or BMI category (‘normal’ BMI <25, ‘overweight’ BMI 25 - 30), therefore daily energy intake by portion size condition is plotted by gender and BMI category for descriptive purposes and shows the same pattern of daily intake between categories (Figure S4).

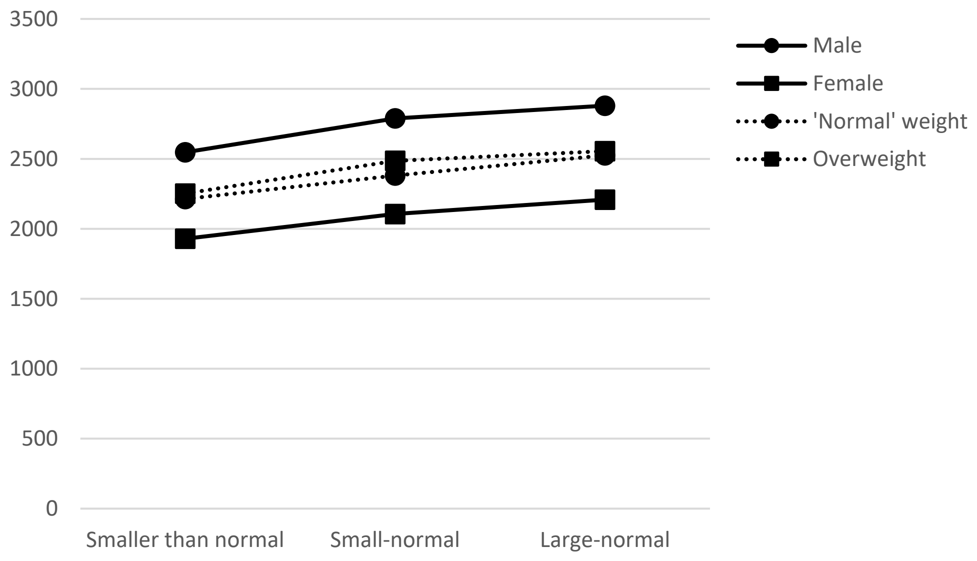

Figure S4. Daily energy intake (kcal) by condition plotted by gender and BMI category.

Table S4.

Mean normality ratings for each meal by portion size (*SD*).

	‘Smaller than normal’	‘Small-normal’	‘Large-normal’
Spaghetti bolognese	2.2 (0.9)*	4.2 (1.0)	5.6 (1.3)*
Spaghetti carbonara	2.4 (1.0)*	4.1 (0.7)	5.8 (1.0)*
Penne pesto	2.1 (0.9)*	4.4 (0.9)	6.2 (1.0)*
Chili con carne	3.0 (1.1)*	4.6 (1.0)*	6.2 (1.1)*
Chicken korma curry	2.4 (1.1)*	4.3 (0.6)	5.7 (0.8)*
Beef curry	2.3 (1.1)*	4.6 (1.0)*	6.3 (0.9)*

*p <0.004 for one-sample t tests comparing with midpoint of the normality rating scale (4 “…how normal is this portion?…”), corresponding to perceived ‘normal’. Midpoint comparison analyses for smaller than normal and small-normal were pre-registered. Large-normal midpoint comparison analyses were requested during peer review. All pairwise comparisons of normality ratings between portion sizes for each of the 6 meals are significant (p<.001).

References

1. Haynes A, Hardman CA, Halford JCG, Jebb SA, Robinson E. Portion size normality and additional within-meal food intake: two crossover laboratory experiments. British Journal of Nutrition. 2019.
2. Van Strien T, Frijters JER, Bergers GPA, Defares PB. The Dutch Eating Behavior Questionnaire (DEBQ) for assessment of restrained, emotional, and external eating behavior. International Journal of Eating Disorders. 1986;5(2):295-315.
3. Robinson E, Aveyard P, Jebb SA. Is plate clearing a risk factor for obesity? A cross-sectional study of self-reported data in US adults. Obesity. 2015;23(2):301-4.
4. Hunot C, Fildes A, Croker H, Llewellyn CH, Wardle J, Beeken RJ. Appetitive traits and relationships with BMI in adults: Development of the Adult Eating Behaviour Questionnaire. Appetite. 2016;105:356-63.
5. De Ridder DTD, de Boer BJ, Lugtig P, Bakker AB, van Hooft EAJ. Not doing bad things is not equivalent to doing the right thing: Distinguishing between inhibitory and initiatory self-control. Personality and Individual Differences. 2011;50:1006-11.
